# What Is Facial Neuralgia?

**Published:** 1882-06

**Authors:** Parsons Shaw

**Affiliations:** Manchester, England.


### ARTICLE IV.

### *What is Facial Neuralgia?*

BY PARSONS SHAW, D. D. S., MANCHESTER, ENGLAND.

I have been requested to report the following case and do so for the sake of its lesson, the names of all the parties, for obvious reasons, being suppressed. A well known dentist in one of our largest cities was called in consultation with the regular medical attendant to see an old patient, a merchant, whose loss would not only be irreparable to his family, but also keenly felt throughout a wide commercial circle. By the express desire of the patient no intimation of his symptoms was conveyed to the dentist, in order, I presume, that he might obtain an entirely unbiased opinion from that gentleman; and he proceeded to state that he had been confined to his house for more than a month, and for some time even to his bed, with what had been pronounced a severe attack of neuralgia, and for which he was then under treatment. He described his pains as mostly confined to one side of the face, but often shooting through the entire face, and frequently extending into the head, neck, back, and even more distant regions. The dentist inquired into the condition of the teeth, and was told, what proved to be true on examination, that they were not at all decayed or sore, but that a little matter had been observed at the edges of the gum. He could not say, at the time, he had noticed any discharge of pus from, or had perceived any fœtid smell in his nose; but afterwards remembered that he had. There was no very perceptible swelling of the face, yet the patient pointed to a sore placc just beneath the eye, and close examination showed it was a little puffed on the side where the pain was mostly felt. He had become very weak, and his whole system was generally deranged. Every intelligent dentist who has read thus far, will have concluded—as did the dentist consulted, in less than five minute's time—that this patient was suffering from a severe attack of inflammation in the lining membrane of the antrum When the regular medical attendant heard this opinion, he at once concurred in it, and also ageed to the desirability of the course of treatment suggested by the dentist for the discharge of the accumulated pus in the antrum. But before he would consent to any operation, he insisted on having the opinion of a certain eminent surgeon. Two days were thus allowed to pass before this gentleman could be got in attendance, the patient's sufferings going on, and the chances of his recovery being greatly reduced in the meanwhile. In time, however, the physician, surgeon and dentist met, when the strong advice of the latter was acted upon, and a molar tooth removed, with the understanding that if this did not procure sufficient discharge of pus from the antrum, an opening was to be made by means of the dental engine. The tooth was removed with very little exertion, and a considerable quantity of matter discharged, but it was evident that little of it came from the antrum. The dentist, in due course of time, took his leave, quite expecting to be again called as soon as the patient had recovered somewhat from the first, to perform the second operation, which all had so far agreed with him to be necessary for the removal of the primary cause of the illness. But, to his great surprise, he was not again consulted, beyond being asked, a few days after the extraction, to determine a dispute which had arisen between a fourth authority—a consulting physician who had been called in—and the attending physician, as to the character of several pieces of necrosed alveolus which the patient had removed with his fingers, the eminent consulting physician having declared it was a part of the tooth which the dentist must have broken in the extraction? As the dentist was not again consulted, he only knows what took place informally, and not from his own observations; but the information is sufficiently accurate for us to come to a definite conclusion.

As the patient got gradually worse, it was given out, as the result of the combined wisdom of his medical advisers—minus the dentist—that he was suffering from a *complication of disorders*, such as neuralgia sciatica, gout in the feet, Bright's disease, etc., as if each of these were idiopathic, accompanied by intermittent febrile symptoms. In about eight or ten days after the removal of the tooth, he had a severe rigor, which was repeated at irregular intervals; and in just fourteen days after the operation, he died. If the dentist has had no difficulty in determining the exciting cause of this patient's disorder, neither will the thoughtful physician be at a loss to understand from what he died. Never did symptoms point more clearly to a case of pyœmia. The pent-up pus which had been allowed to accumulate in the antrum for more than a month before the cause of his symptoms was discovered by his dentist, had, in all probability, wrought sufficient mischief to render the patient's recovery doubtful; the more especially as the alveolus was found to be so much necrosed as to come away in large pieces; and albuminuria had already developed. Yet, if the antrum had been opened, as was advised, and the matter removed, and an antiseptic treatment adopted, there was still a fair chance of recovery, as the patient lived two weeks after the removal of the tooth ; and while there were febrile symptoms, there was no rigor for some time. whereas, death from blood poisoning, results, as a rule, in from four to ten days. The possibility of recovery was also heightened from the fact that the patient had a good constitution and enjoyed excellent health. Yet so prepossessed were the minds of those who were responsible for this life, that they were apparently not able to make out a correct diagnosis, even after the key to the situation had been placed in their hands by the dentist, and the patient was left to linger on in pain, and be finally lost to all his usefulness, except that of allowing me to point out the error which led to such a catastrophe.

It is not pleasant to look back on a case like this, and nothing but a strong sense of duty, and the conclusion to which it unmistakably points, could induce me to do so. Is it not high time that dentists took this "facial neuralgia" theory in hand and exposed its errors, and, as we have seen, sometimes fatal consequences? It is not diagnosis, but pure charlatanism, to tell a patient who consults a medical man for the relief of a facial pain, that he has a "nerve pain." He knew that quite well enough, and what he wants is relief. But it is not proceeding towards this desirable end to assume that a pain cannot better be defined than by the use of a term the patient does not, as a rule, understand ; or if he does, concludes it is one of those general terms, which has, by common consent, become crystalized into a definite meaning, which is well understood by those who use it ; that his symptoms are his disease ; and by the confident manner in which he is thus assured, lead him to believe that, even if he does not understand the meaning of neuralgia, the doctor knows all about his case. It is a common occurrence to find patients treated for weeks, and even months, for neuralgia, while any intelligent dentist would, from the patient's description of the symptoms alone, at once say they arose from either odontitis or periodontitis; or, failing these, inflammation of the lining membrane of the antrum, and this apparently without a suspicion on the part of the medical attendant that the pain had any such origin. Even when alveolar abscess has caused the face to swell, I have known men, justly regarded as eminent physicians, to treat the patient for erysipelas. And, also, when the pus had been induced to approach the surface by means of powerful irritants, to lance the face in order that it might discharge in that direction, and then declare it a case of scrofula. There can be nothing without a cause; yet, so-called facial neuralgia is regularly treated as if it arose without any definite cause, or from some general or remote cause, which was quite too obscure for anything like accurate diagnosis, while in nine cases out of ten the origin of the pain so described stares one in the face the moment the mouth of the patient is examined; and almost invariably is only obscure to inexperienced or superficial observation. So true is this, that to the astute dentist no meaning will be attached to such a term as facial neuralgia, for he knows that if the pains do not arise from some lesion in or around the teeth, or in the antrum, the origin may then be looked for deep in the neural axis; in which case the patient is in a condition to demand the most anxious attention of his medical adviser and nearest friends. Some years since I had just such a case in my own practice. The patient complained of pains in his head and face after I had made certain they could not arise from his teeth. When he applied to anyone else for advice he was assured he had "neuralgia," and my suggestion that his was in some way affected, received no encouragement from the physicians he consulted, simply because they regarded his pains as an ordinary form of what they taught him was a well-defined complaint. In time he died so suddenly that a post-mortem examination was required, and I was ordered by the coroner to attend it, when, after much search, the pineal gland was found a mass of disorganized matter. The erratic pains were then accounted for by some disturbance in the reflection of nerve irritation.

Such cases as are here named might be repeated by the score by any observant dentist. Yet we allow them to go on, and the general practitioner to assume superior knowledge, where we should always teach.—*Missouri Dental Journal.*

